# Association between Access to Public Open Spaces and Physical Activity in a Mediterranean Population at High Cardiovascular Risk

**DOI:** 10.3390/ijerph15061285

**Published:** 2018-06-17

**Authors:** Antoni Colom, Miguel Fiol, Maurici Ruiz, Montserrat Compa, Marga Morey, Manuel Moñino, Dora Romaguera

**Affiliations:** 1 Instituto de Investigación Sanitaria Illes Balears (IdISBa), University Hospital Son Espases, Palma de Mallorca 07120, Spain; antonicolom@gmail.com (A.C.); miguel.fiol@ssib.es (M.F.); marga.morey@yahoo.es (M.M.); mmonyino@gmail.com (M.M.); 2 CIBER Fisiopatología de la Obesidad y Nutrición (CIBEROBN), Instituto de Salud Carlos III, Madrid 28029, Spain; 3 Servicio de SIG y Teledetección, Vicerectorat d’Innovació i Transferència, Universitat de les Illes Balears, Palma de Mallorca 07120, Spain; maurici.ruiz@uib.es; 4 Instituto Español de Oceanografía, Centro Oceanográfico de Baleares, 07015 Palma, Spain; montserratcompa@gmail.com

**Keywords:** physical activity, leisure, public open space, GIS, elderly, PREDIMED trial

## Abstract

*Background*: Regular physical activity is an important preventive factor of cardiovascular disease. Proximity and density of public open spaces are important modifying factors on the practice of physical activity. This article explores the cross-sectional relationship between access to public open spaces (POS) and leisure time physical activity (LTPA) in elderly participants at high cardiovascular risk from PREDIMED-Baleares. *Method*: 428 elderly subjects at high cardiovascular risk, participating in the PREDIMED trial, from Palma de Mallorca (Spain) were assessed using Geographic Information Systems, and access to POS was determined. The quantity and intensity of LTPA was calculated using the Minnesota Leisure-Time Physical Activity Questionnaire. In order investigate the association between access to POS and LTPA, generalized linear regression models were used. *Results*: Better access to POS was not consistently associated with total LTPA. Only distance to the nearest park showed a borderline significant positive associated with total LTPA and moderate-vigorous LTPA but was not associated with light LTPA. *Conclusions*: Although living near POS was not associated to total LTPA, higher levels of moderate-vigorous LTPA were associated to distances to the nearest park. Future work should be conducted on a larger sample size, integrating a longitudinal design, and greater heterogeneity in POS access and introducing objective measures of physical activity.

## 1. Introduction

Cardiovascular disease is one of the leading causes of death worldwide [1] with one of the primary reducers of the risk of cardiovascular disease (CVD) being physical activity [2]. Several recent studies have shown inactive individuals are twice as likely to be at risk of CVD compared to active individuals [3]. In addition, regular physical activity has shown to reduce many of the risk factors for CVD, including hypertension, obesity, and other chronic disorders [4].

Physical activity has not only shown to have biological implications improving overall health, but also social implications providing with a more active and independent lifestyle [5]. However, despite this, the elderly age group still remains among the least active of all age groups. According to Edwards and Tsoaris, 60% of Europeans older than 65 years did not engage in any moderate physical activity [6].

Built environment may be an important determinant of physical activity practice in this population. Public open spaces (POS) such as parks, beaches or sport facilities are key built environment settings for physical activity and can influence physical activity in at least three ways: as a setting to engage in physical activity, promoting active travel or to socialize [7]. In some studies, the availability of and the proximity to recreational facilities has been associated consistently with greater physical activity among adults [8,9]. Since POS are generally accessible to large populations, they are well positioned as a mass influence to act on the population as a whole in order to play a role in disease prevention [10,11]. In addition, different type of POS may have different associations with physical activity. For instance, there is some evidence that non-park public open space might be important for physical activity [7,12]. Therefore, it is important to study not only overall POS access, but also different type of POS. The interest in the relationship between the built environment and physical activity levels among the elderly population has grown steadily over the past years, resulting in a substantial increase in studies on this topic across the world [13]. In general, studies of the accessibility of public open spaces (POS) and their influence in physical activity are predominantly from Australia, UK and USA [14,15,16,17,18,19,20,21], while in the Mediterranean setting, this topic has been the subject of a limited number of studies [22]. This has resulted in a gap of information and the need to investigate within the Mediterranean context separately which different types of POS are associated to physical activity since low CVD risk and longevity has often been associated to the Mediterranean life style without taking into account intrinsic factors such as their surrounding environment.

The objective of the present study was to explore the cross-sectional association between access to POS, specifically the resources that may be relevant to our population: sports facilities, parks and beaches, and the practice of leisure time physical activity (LTPA) among a subsample of elderly subjects at high cardiovascular risk participating in the PREDIMED-Baleares trial, a randomized dietary primary prevention trial conducted in the city of Palma de Mallorca, Balearic Islands, Spain. To achieve this, we aim to: (i) examine the exposure to each type of POS for each participant with the use of Geographic Information Systems (GIS) and (ii) assess whether self-reported physical activity, measured with validated questionnaires, is associated with accessibility to each type of POS.

## 2. Materials and Methods

### 2.1. Study Population

The PREDIMED study was designed to evaluate the effect of an intervention promoting the consumption of a Mediterranean diet, supplemented with extra-virgin olive oil or nuts, compared to a control low-fat diet, for the primary prevention of cardiovascular disease (CVD). No advice on physical activity was given to participants. Participants were women aged 60 to 80 and men aged 55 to 80 enrolled from primary care facilities in Spain between 2003–2009 who were at high cardiovascular risk but without previous CVD. They had type 2 diabetes mellitus or at least three of the following major cardiovascular risk factors: smoking, hypertension, elevated low-density lipoprotein cholesterol levels, low high-density lipoprotein cholesterol levels, overweight or obesity, or a family history of premature coronary heart disease. The full design of the trial has been described elsewhere [23,24].

The Balearic Islands was one of the 11 recruitment centers participating in the PREDIMED study. The study protocol and ethical approval was granted by the Hospital Son Dureta institutional review boards and all participants provided written informed consent.

**Figure 1:**
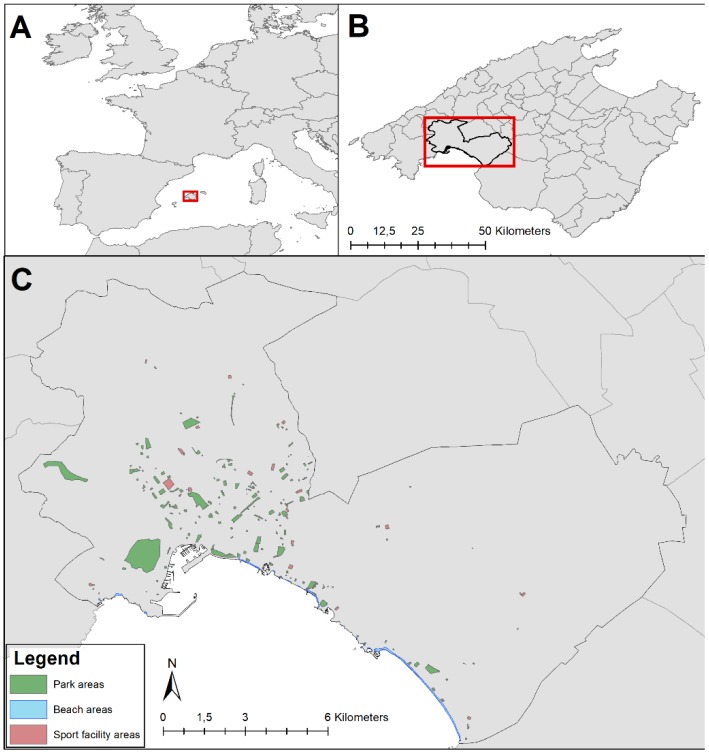
Study area of city of Palma de Mallorca, with location of the Public Open Spaces: parks (green), beach (blue), sport facility (red).

A total of 455 participants enrolled in the PREDIMED-trial in the Balearic Islands who reported living within the city limits of Palma de Mallorca were selected (Figure 1). We excluded 27 participants without registered physical activity at baseline. A final sample of 428 participants was used for the analysis.

### 2.2. Measure of Exposure to Public Open Spaces (POS)

The first step was to create the POS data layer from three different data sources: sports facilities from the Municipal Sports Institute of the City of Palma; beaches from the Balearic Islands Coastal Observartion and Forecasting System; parks from the Department of Infrastructure and Accessibility of the City Council of Palma. Sports facilities included only public facilities owned and maintain by the city council relevant to our population: multi-sports centers, gyms, covered and non-covered swimming pools, indoors and outdoors courts (basketball, tennis, football), and bocce ball ranks. All privately run sports facilities (golf courses, private gyms or private courts) were excluded from the definition, because their access is neither open nor public. The proximity to the coast as a potential resource for practice LTPA was included [25], since the city of Palma is a coastal city.

Secondly, we geocoded participants’ resident addresses reported at baseline, using the Web Processing Service provided by the CartoCiudad project run by the National Geographic Institute of Spain, which is freely available for download (www.cartociudad.es).

**Figure 2:**
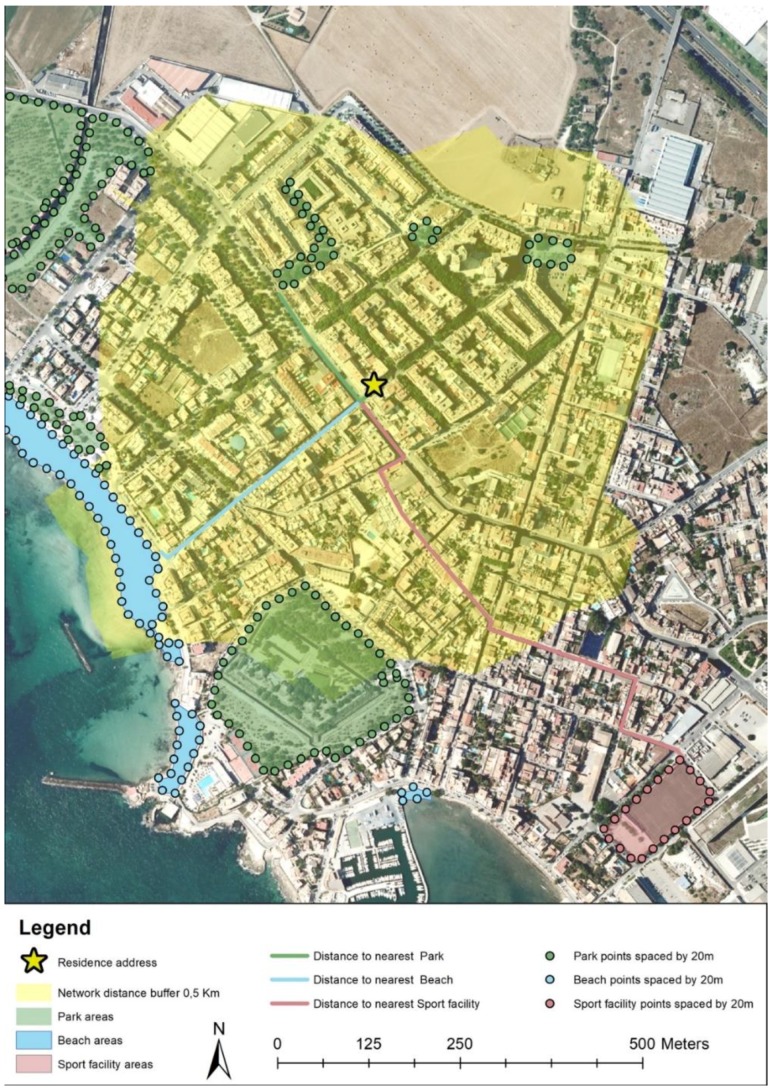
Example of exposure to public open spaces (POS). A 0.5 km network walkable street buffer around of the residence address represented by the yellow area.

Finally, the POS data layer for the City of Palma was used to calculate three objective indicators of exposure: the distance of the walkable street network to the closest POS, the number of POS and the total area of POS within each network walkable street buffers (0.5 km, 1 km and 1.5 km) of participants residential addresses, using only the walking and/or cycling street network, ignoring routes restricted to pedestrians such as freeways, adapting recommendations from previous studies [26] (Figure 2). Due to the existence of streets and an absence of pedestrian foot paths and sidewalks, walkable network street buffers were used [27,28].

Walkable network street buffers were generated using the Service Area Layer within the Network Spatial Analyst extension of ArcGIS (v.10.2, ESRI, Redlands, CA, USA). The function calculates a walking and cycling street network region that encompasses all streets that can be accessed within a given distance from the participant’s resident addresses.

#### 2.2.1. Proximity to the Nearest POS

For the purpose of better assessing distance to the closest POS, we transformed the boundaries of each polygon to points spaced every 20 m from each other. In the absence of POS entry points this, method allows us to eliminate the measurement error caused by the shape and size of the resource if we used the centroid of the POS [29]. Distances to the closest POS boundary point were identified using the Origin-Destination Cost Matrix function within the Network Spatial Analyst extension of ArcGIS. The function calculates a walking and cycling street network distance from the participant’s resident addresses to a nearest POS boundary. We considered distance to each type of POS separately (sports facilities, beaches and parks) as well as distance to the coast. We did not consider distance to any POS as an exposure because for 85.5% of the sample the nearest POS is a park.

#### 2.2.2. Density of POS: Number of POS and Total Area of POS.

We used the tabulate intersection tool to compute the intersection between each street walkable network buffers and each POS. A spatial join was used in order to calculate the total area and count of POS within or partially within the network walkable street buffers of 0.5 km, 1 km and 1.5 km for each participant.

### 2.3. Outcome Measure: Leisure Time Physical Activity

Leisure time physical activity (LTPA) was assessed at baseline using the validated Spanish version of the Minnesota Leisure-Time Physical Activity Questionnaire [30,31], with the intention of calculating the amount of energy expended as LTPA. The questionnaire is composed of 67 activities, including two specific questions about active travel to work (walking or cycling), two specific questions about non-work active travel as walking to and from the shops (carrying shopping cart or bags), as well as a question about leisure walk, and has been validated for men and women as it is described extensively elsewhere [30,31]. Number of days and min/day of activities practiced during the previous week and year during leisure time was asked in face-to-face interviews with trained dietitian. Metabolic equivalent task in minutes per day (METs min/day) has been obtained by multiplying the METs assigned to each leisure time activity for the time (minutes per day) of activity.

Initially LTPA was classified as light (intensity below 4 METs), moderate (intensity 4–5.5 METs) and vigorous (intensity greater than or equal 6 METs) but given that this population reported low moderate and vigorous intensities of leisure time physical activities with a medians and interquartile ranges of 8 (0–40.1) and 0 (0–108) METs·min/day respectively, we decided to combine moderate and vigorous physical activity (MVPA) into one category.

### 2.4. Covariate and Confounders Assessment

Covariables were assessed using the eligibility questionnaire and general questionnaire, including several questions on sociodemographic status and overall health. Of those, we selected as plausible confounders, those that based on previous knowledge [32,33,34] were associated with the exposure or outcome of interest: sex, age, body mass index, educational level (as a proxy of socio-economic status) and smoking habits at baseline [23]. Body mass index was calculated as a participant’s weight in kilograms divided by the square height in meters. Educational level was coded as six levels variable, which we grouped in three levels: secondary school or higher; primary school or less and insufficient data. Information about smoking habits was grouped in three levels: smoker, ex-smoker and never smoker.

### 2.5. Statistical Analysis

A descriptive analysis was performed comparing participant general demographic characteristics and the POS variables according to the categories of total LTPA, based on the tertile distribution. The Compare Groups package for R [35] was used to determine the significance of the trend. Continuous normally distributed baseline characteristics were compared using Pearson test and continuous non-normally distributed baseline characteristics were compared using Spearman test. Finally, and categorical baseline characteristics were compared using chi-squared.

In a second step, we examined the influence of POS on the total LTPA, moderate-vigorous LTPA and light LTPA. For this purpose, a generalized linear regression was used. We constructed a baseline model (Model 1) and a second model adjusted for each participant’s individual-level plausible confounders (Model 2): sex, age, body mass index, educational level (as a proxy of socio-economic status), and smoking habits. Due to the multiple comparisons performed, we applied the Bonferroni post-hoc correction on both models. These analyses were performed using fitting Generalized Linear Models and Adjust *p*-values for Multiple Comparisons in the R Stats Package 3.3.3 [36].

## 3. Results

In our study, men had a higher total LTPA than women; nonetheless 54.2% (42.5% men and 57.3% women) of the population did not meet the minimum recommended level of moderate-vigorous LTPA of 500 METs·min/week (data not shown in tables). The median and interquartile range of total LTPA was 198 (3.8 to 330) METs·min/day; of light and moderate-vigorous LTPA was 87.9 (14.0 to 176) METs·min/day and 49.5 (6.03 to 179) METs·min/day, respectively (Table 1). Results for each POS (parks, beaches, sports facilities and coastal proximity) separately are presented as supplementary material (Supplementary Table S1).

Participants in the highest tertile of total LTPA were more likely to be older, had lower BMI and were less likely to be current smokers (Table 1). No significant association between tertiles of LTPA and educational level were found.

Table 2 shows the mean values for each of the possible POS scenarios, by tertiles of LTPA. Overall, non-significant associations were found between tertiles of total LTPA and access to POS, except for a borderline significant association between distance to the nearest park and total LTPA (i.e., participants with lower level of LTPA had their place of residence closer to the nearest park).

Table 3 shows the association between total LTPA and distance to POS, sums of areas and counts within each network walkable street buffers of 0.5 km. Results for 1 km and 1.5 km buffers are shown in the Supplementary Tables S1 and S2. Both unadjusted (Model 1) and adjusted (Model 2) by individual-level covariates models are displayed. Overall no statistically significant associations were observed, except for a positive significant association in the unadjusted Model 1 between distance to the nearest park and total LTPA, that lost statistical significance to a borderline significant association on the Model 2 adjusted by individual-level covariates and after Bonferroni correction. When the models were run separately for each POS (parks, beaches, sports facilities and coastal proximity) for total LTPA (Supplementary Table S2), for light LTPA (Supplementary Table S3) and for moderate-vigorous LTPA (Supplementary Table S4) we observed similar results (no significant associations), however the positive association between distance to the nearest park and moderate-vigorous LTPA presented positive statistically significant association in the Model 1 unadjusted and Model 2 adjusted by individual-level covariates: increments of 100 m of distance to the near park, were associated with an increase of 17.77 METs·min/day of moderate-vigorous LTPA using the adjusted model (Supplementary Table S4). 

## 4. Discussion

The purpose of the present study was to examine the association between access to POS and LTPA across older adults at high cardiovascular living in a Mediterranean city than on average showed low levels of LTPA. Overall, no association was detected between access to POS and LTPA. In our results, distance to the nearest park showed a borderline significant positive associated with total LTPA and moderate-vigorous LTPA.

In general, studies predominantly from Australia, UK and USA, have demonstrated that living in closer proximity to POS was associated with a greater likelihood of being physically active and improved health, for all age groups [14,15,16,17,18]; however, other studies have shown null or weak associations between objective measures of availability of POS and LTPA [17,19,20,21].

For instance, Rutt and Coleman (2005) found no associations between built environment and self-reported physical activity during the last month, but the exception was that vigorous physical activity increased when people lived further from physical activity facilities such as parks, gyms, schools, and biking/walking paths [20]. However other studies have reported the contrary, for instance, Thornton et al. (2017) found a negative association between GIS-measured distance of the closest park and MVPA [33]. Michael et al. (2010) found that distances to a park was positively associated with maintaining or increasing walking between baseline and after an average of 3.6 years of follow-up, although neither reached statistical significance [37]. Differences in results between our study and previous studies could be due, among others, to the different methodologies and criteria used to define POS and physical activity. However, it is important to make an appreciation of cultural diversity. In particular, Mediterranean populations are nowadays characterized for being more sedentary. According to the Global Health Observatory (WHO 2010), prevalence of insufficient physical activity was highest in the Mediterranean Region; concretely in Spain 33.7% of women and 27.2% of men were insufficiently active. Also, our study includes a homogeneous sample of elderly population at high cardiovascular risk, which may also be more sedentary than the average population.

This study has some strengths: first, contrary to previous studies, this study is conducted in a Mediterranean population that has been subjected to a limited number of studies on this topic, despite its singularities. Second, because of the use of a quantitative and objective measures of POS (as opposed to participant self-report) our results are not subjected to same-source bias that might arise in survey-only studies (i.e., physically active participants being more likely to report resources in their local area) [38]. Also, we evaluated a wide range of POS, including beach access and distance to the coast (where there is a walking and biking path), variables that are relevant to our population and may influence LTPA. We used walkable street buffers, instead of radial buffer in order to minimize measurement errors. Additionally, although our study used self-reported LTPA and sedentary time which may be subject to potential biases, we used specifically validated questionnaires [30,31].

Like previous studies using empirical data, this study also has a few limitations. The first is the low sample size, which although sufficient for this particular study, it was not large enough to perform stratification analyses by age group, sex or educational level, factors that may have modified the association; hence, future work would benefit from a higher sample size. Second, the population under study is a homogeneous sample of older participants at high cardiovascular risk, with overall low physical activity levels, from a medium size city in the Mediterranean. This homogeneity may have led to low variability in the access to POS, which may have limited our ability to observe significant associations between the access to POS and LTPA. The results might not be generalizable to older adults other than those living in urban areas in the City of Palma. Our data on physical activity, despite coming from a validated questionnaire, did not allow us to differentiate the types of activity that may have been relevant to our exposures, i.e., walking or gym workout. On the other hand, our data on access to POS was only objectively measured being impossible to identify the perceived preferences over the POS. In addition, we did not take into account the size of the POS and it could be possible that small resources that may not be relevant for PA have been included in our analyses. The interest in this study was on public open spaces; yet, we cannot rule out that other resources for PA practice (i.e., privately owned) may be important in this population. Another limitation in this study is its cross-sectional study design; therefore, we cannot infer causality or establish that the exposures preceded the outcomes, hence we cannot use these results to evaluate their policy implications. Nevertheless, cross-sectional studies are important for expediently identifying factors that might be targeted to improve LTPA in future longitudinal or community intervention studies. Finally, as with any observational study, residual confounding cannot be ruled out, despite our effort to control for confounders in our models.

## 5. Conclusions

Although living near POS was not associated to total LTPA, distances to the nearest park were found to be associated to increased levels of moderate-vigorous LTPA among older adults living in the city of Palma. Future work should be conducted on a larger sample size, integrating a longitudinal design, and greater heterogeneity in POS access with objective measures of physical activity.

## Figures and Tables

**Table 1:** Study population demographic characteristics according to tertiles of total leisure time physical activity (LTPA).

Individual/Demographic	Total Leisure Time Physical Activity (LTPA)				
	(ALL) *n* = 428	T1 *n* = 143	T2 *n* = 142	T3 *n* = 143	*p* Trend
Sex					<0.001
Men	182 (42.5%)	49 (34.3%)	51 (35.9%)	82 (57.3%)	
Women	246 (57.5%)	94 (65.7%)	91 (64.1%)	61 (42.7%)	
Age (years)	68.0 (63.0;72.0)	67.0 (62.0;71.0)	68.0 (63.0;72.0)	69.0 (65.0;73.0)	0.016
BMI (kg/m^2^)	30.0 (27.5;32.1)	30.9 (28.3;33.0)	29.7 (27.4;31.8)	29.9 (27.2;31.6)	0.012
Educational Level					0.159
Primary school or lesser	365 (85.9%)	125 (87.4%)	125 (88.7%)	115 (81.6%)	
Secondary School or greater	60 (14.1%)	18 (12.6%)	16 (11.3%)	26 (18.4%)	
Smoking status					0.008
Former	118 (27.6%)	31 (21.7%)	28 (19.7%)	59 (41.3%)	
Current	70 (16.4%)	31 (21.7%)	25 (17.6%)	14 (9.79%)	
Never	240 (56.1%)	81 (56.6%)	89 (62.7%)	70 (49.0%)	
Total LTPA (METs min/day)	198 (93.8;330)	50.1 (14.6;93.7)	198 (160;226)	432 (330;573)	<0.001
Light LTPA (METs min/day)	87.9 (14.0;176)	28.0 (0.00;70.0)	140 (76.0;173)	177 (44.4;298)	<0.001
Moderate-vigorous LTPA (METs min/day)	49.5 (6.03;179)	8.00 (0.00;24.0)	57.6 (8.01;132)	277 (123;455)	<0.001

Values shown are *n* (%) for categorical variables and median (IQR) for continuous variables. Tertile cutoffs are based on total leisure time physical activity (LTPA). The *p*-value for trend is computed from the Pearson test when row-variable is normal, Spearman test when it is continuous non-normal and Chi-squared test when it is categorical.

**Table 2:** Study population POS characteristics according to tertiles of total leisure time physical activity (LTPA). Values shown are *n* (%) for categorical variables and median (IQR) for continuous variables. Tertile cutoffs are based on total LTPA. The p-value for trend is computed from the Pearson test when row-variable is normal, Spearman test when it is continuous non-normal and Chi-squared test when it is categorical.

	Total Leisure Time Physical Activity (LTPA)				
	(All) *n* = 428	T1 *n* = 143	T2 *n* = 142	T3 *n* = 143	*p* trend
Distance to the nearest sports facility (m)	460 (289;724)	405 (240;652)	499 (301;744)	456 (319;692)	0.191
Distance to the nearest Park (m)	187 (113;292)	172 (104;287)	174 (106;266)	213 (132;314)	0.041
Distance to the nearest beach (m)	2951 (2316;5119)	3220 (2313;5239)	3064 (2275;5263)	2838 (2371;4842)	0.202
Distance to the coast (m)	2710 (1676;4037)	2868 (1737;4044)	2667 (1952;4184)	2584 (1519;3779)	0.399
Sum areas in 500 m^2^ network walkable street buffers (m^2^)	13,376 (6023;20317)	14,499 (7840;20416)	12,795 (6321;20005)	11,792 (5431;20561)	0.062
Sum counts in 500 m^2^ network walkable street buffers	4.00 (3.00;5.00)	4.00 (3.00;5.00)	4.50 (3.00;5.00)	4.00 (3.00;6.00)	0.530

Abbreviations: POS, public open spaces; LTPA, leisure time physical activity.

**Table 3:** Summary of the results from the generalized linear regression (GLM) for the association between access to Public Open Spaces (POS) and total leisure time physical activity (LTPA). The results for the following model comparisons are provided: unadjusted GLM (Model 1) GLM adjusted by individual-level covariates (sex, age, body mass index, educational level and smoking habits). Due the use of multiple comparisons we applied the Bonferroni post-hoc correction on both model.

Predictor Variable	Model 1			Model 2		
	ß	CI	*p*	ß	CI	*p*
Distance to the nearest sports facility (per 100 m)	5.724	(1.134; 10.314)	0.149	4.462	(0.066; 8.859)	0.473
Distance to the nearest Park (per 100 m)	16.66	(6.996; 26.324)	0.008	13.393	(4.2; 22.585)	0.045
Distance to the nearest beach (per 100 m)	−0.559	(−1.756; 0.638)	1.000	−0.622	(−1.767; 0.522)	1.000
Distance to the coast (per 100 m)	−0.216	(−1.551; 1.119)	1.000	−0.472	(−1.759; 0.815)	1.000
Sum areas in 500 m^2^ network walkable street buffers (per 100 m^2^)	−0.121	(−0.316; 0.073)	1.000	−0.089	(−0.273; 0.095)	1.000
Sum counts in 500 m^2^ network walkable street buffers	−5.952	(−16.5; 4.596)	1.000	−3.626	(−13.607; 6.355)	1.000

Abbreviations: ß, non-standardized coefficient; CI, confidence interval; *p*, p-value for trend. ß indicates change in total LTPA Metabolic equivalent task per minutes per day (METs min/day) per increment (in 100 m, 100 m^2^ or count) in access to public open spaces (POS). Model 1: unadjusted linear regression. Model 2: linear regression adjusted by individual-level covariates (sex, age, body mass index, educational level and smoking habits). Due the used of multiple comparisons we applied the Bonferroni post-hoc correction on both model.

## Supplementary Materials

The following are available online at http://www.mdpi.com/1660-4601/15/6/1285/s1, Table S1: Study population POS characteristics according to tertiles of total LTPA, Table S2: Association between access to POS and total LTPA, Table S3: Association between access to POS and light LTPA, Table S4: Association between access to POS and moderate-vigorous LTPA.

## Declaration

The data that support the findings of this study are available from the PREDIMED study but restrictions apply to the availability of these data, which were used under license for the current study, and so are not publicly available.
