# Supplementary material for: Association between Access to Public Open Spaces and Physical Activity in a Mediterranean Population at High Cardiovascular Risk
Source: Int J Environ Res Public Health. 2018 Jun 17;15(6):1285. doi: 10.3390/ijerph15061285 (PMC6025464; doi:10.3390/ijerph15061285)
Supplement: Supplementary file 1 [file ijerph-15-01285-s001.pdf]

International Journal of
Environmental Research and Public Health

MDPI

Table S1. Study population POS characteristics according to tertiles of total LTPA.

Individual/demographic	Total leisure time physical activity (LTPA)				
	(ALL) N=428	T1 N=143	T2 N=142	T3 N=143	p.trend
Sum areas in 500m2 network walkable street buffers (m2)	13376 (6023;20317)	14499 (7840;20416)	12795 (6321;20005)	11792 (5431;20561)	0.062
Areas of sports facilities in 500m² network walkable street buffers (m²)	665 (0.00;2127)	980 (0.00;2765)	302 (0.00;2231)	665 (0.00;1873)	0.220
Areas of parks in 500m² network walkable street buffers (m²)	10921 (4540;16731)	12054 (4855;16808)	10921 (5270;15466)	8372 (3813;17798)	0.181
Areas of beaches in 500m² network walkable street buffers (m²)	0.00 (0.00;0.00)	0.00 (0.00;0.00)	0.00 (0.00;0.00)	0.00 (0.00;0.00)	0.808
Sum areas in 1000m2 network walkable street buffers (m2)	55884 (25371;84564)	52982 (23885;82765)	52497 (26012;82383)	62346 (38159;85484)	0.160
Areas of sports facilities in 1000m² network walkable street buffers (m²)	6169 (1908;12147)	6173 (1908;12183)	5521 (2018;10298)	7221 (1908;12241)	0.692
Areas of parks in 1000m² network walkable street buffers (m²)	47316 (17082;74754)	41194 (15059;72580)	45234 (15877;72910)	51507 (20157;76383)	0.156
Areas of beaches in 1000m² network walkable street buffers (m²)	0.00 (0.00;0.00)	0.00 (0.00;0.00)	0.00 (0.00;0.00)	0.00 (0.00;0.00)	0.892
Sum areas in 1500m2 network walkable street buffers (m2)	142933 (70527;192218)	140579 (67774;185053)	141272 (66993;190953)	148957 (76845;197901)	0.188
Areas of sports facilities in 1500m² network walkable street buffers (m²)	14224 (8949;26326)	14196 (6171;28195)	13920 (8940;26794)	16543 (9578;22848)	0.783
Areas of parks in 1500m² network walkable street buffers (m²)	101252 (31410;174050)	95581 (23823;162574)	93928 (32815;173559)	120737 (59315;182119)	0.143
Areas of beaches in 1500m² network walkable street buffers (m²)	0.00 (0.00;0.00)	0.00 (0.00;0.00)	0.00 (0.00;0.00)	0.00 (0.00;0.00)	0.808
Sum counts in 500m2 network walkable street buffers	4.00 (3.00;5.00)	4.00 (3.00;5.00)	4.50 (3.00;5.00)	4.00 (3.00;6.00)	0.530
Count of sports facilities in 500m² network walkable street buffers	1.00 (0.00;1.00)	1.00 (0.00;1.00)	1.00 (0.00;1.00)	1.00 (0.00;1.00)	0.404
Count of parks in 500m² network walkable street buffers	3.00 (2.00;4.25)	3.00 (2.00;4.00)	3.00 (2.00;5.00)	3.00 (2.00;5.00)	0.894
Count of beaches in 500m² network walkable street buffers	0.00 (0.00;0.00)	0.00 (0.00;0.00)	0.00 (0.00;0.00)	0.00 (0.00;0.00)	0.775
Sum counts in 1000m² network walkable street buffers	12.0 (7.00;17.0)	12.0 (6.00;16.0)	11.0 (7.00;16.0)	14.0 (7.50;17.0)	0.087

Count of sports facilities in 1000m² network walkable street buffers	2.00 (1.00;3.00)	2.00 (1.00;3.00)	2.00 (1.00;3.00)	2.00 (1.00;3.00)	0.593
Count of parks in 1000m² network walkable street buffers	10.0 (4.00;14.0)	9.00 (3.00;13.0)	9.00 (4.00;13.0)	11.0 (4.00;15.0)	0.086
Count of beaches in 1000m² network walkable street buffers	0.00 (0.00;0.00)	0.00 (0.00;0.00)	0.00 (0.00;0.00)	0.00 (0.00;0.00)	0.903
Sum counts in 1500m² network walkable street buffers	20.0 (10.0;32.0)	19.0 (9.50;30.0)	19.0 (10.2;31.8)	23.0 (15.0;32.0)	0.153
Count of sports facilities in 1500m² network walkable street buffers	3.00 (2.00;6.00)	3.00 (2.00;6.00)	4.00 (2.00;6.00)	4.00 (2.00;7.00)	0.200
Count of parks in 1500m² network walkable street buffers	16.0 (7.00;25.0)	16.0 (7.00;24.0)	15.0 (7.00;25.0)	19.0 (11.0;25.0)	0.216
Count of beaches in 1500m² network walkable street buffers	0.00 (0.00;0.00)	0.00 (0.00;0.00)	0.00 (0.00;0.00)	0.00 (0.00;0.00)	0.766

Abbreviations: POS, public open spaces; LTPA, leisure time physical activity. Values shown are n (%) for categorical variables and median (IQR) for continuous variables. Tertile cutoffs are based on total leisure time physical activity (LTPA) The p-value for trend is computed from the Pearson test when row-variable is normal, Spearman test when it is continuous non-normal and Chi-squared test when it is categorical.

Table S2. Association between access to POS and total LTPA.

Predictor variable	Model 1			Model 2		
	ß	CI	¶ P	ß	CI	¶ P
Sum areas in 500m2 network walkable street buffers (per 100m2)	-0.121	(-0.316;0.073)	1.000	-0.089	(-0.273 ; 0.095)	1.000
Sports facility areas in 500m² network walkable street buffers (100m²)	-0.25	(-0.849;0.349)	1.000	-0.316	(-0.888 ; 0.256)	1.000
Parks areas in 500m² network walkable street buffers (100m²)	-0.108	(-0.325;0.109)	1.000	-0.047	(-0.254 ; 0.16)	1.000
Beaches areas in 500m² network walkable street buffers (100m²)	-0.06	(-0.57;0.45)	1.000	-0.128	(-0.61 ; 0.354)	1.000
Sum areas in 1000m2 network walkable street buffers (per 100m2)	0.02	(-0.039;0.078)	1.000	0.023	(-0.034 ; 0.079)	1.000
Sports facility areas in 1000m² network walkable street buffers (100m²)	0.037	(-0.194;0.269)	1.000	0.003	(-0.217 ; 0.224)	1.000
Parks areas in 1000m² network walkable street buffers (100m²)	0.014	(-0.048;0.076)	1.000	0.022	(-0.037 ; 0.082)	1.000
Beaches areas in 1000m² network walkable street buffers (100m²)	0.065	(-0.149;0.278)	1.000	0.031	(-0.171 ; 0.233)	1.000
Sum areas in 1500m2 network walkable street buffers (per 100m2)	0.002	(-0.024;0.028)	1.000	0.007	(-0.019 ; 0.032)	1.000
Sports facility areas in 1500m² network walkable street buffers (100m²)	-0.047	(-0.173;0.078)	1.000	-0.038	(-0.157 ; 0.082)	1.000
Parks areas in 1500m² network walkable street buffers (100m²)	0.004	(-0.024;0.031)	1.000	0.009	(-0.018 ; 0.036)	1.000
Beaches areas in 1500m² network walkable street buffers (100m²)	0.029	(-0.118;0.176)	1.000	0.008	(-0.132 ; 0.147)	1.000
Sum counts in 500m2 network walkable street buffers	-5.952	(-16.5;4.596)	1.000	-3.626	(-13.607 ; 6.355)	1.000
Sports facility counts in 500m² network walkable street buffers	-21.336	(-49.471;6.798)	1.000	-16.764	(-43.622 ; 10.093)	1.000
Parks counts in 500m² network walkable street buffers	-5.804	(-17.386;5.778)	1.000	-2.548	(-13.545 ; 8.448)	1.000
Beaches counts in 500m² network walkable street buffers	14.007	(-14.973;42.987)	1.000	5.953	(-21.543 ; 33.45)	1.000
Sum counts in 1000m2 network walkable street buffers	0.225	(-3.467;3.918)	1.000	0.447	(-3.077 ; 3.972)	1.000
Sports facility counts in 1000m² network walkable street buffers	-3.494	(-17.523;10.535)	1.000	-1.693	(-14.991 ; 11.606)	1.000
Parks counts in 1000m² network walkable street buffers	-0.02	(-4.27;4.229)	1.000	0.319	(-3.738 ; 4.377)	1.000
Beaches counts in 1000m² network walkable street buffers	8.893	(-6.933;24.719)	1.000	5.89	(-9.101 ; 20.88)	1.000
Sum counts in 1500m2 network walkable street buffers	-0.101	(-1.954;1.752)	1.000	0.24	(-1.541 ; 2.022)	1.000
Sports facility counts in 1500m² network walkable street buffers	-0.538	(-7.567;6.491)	1.000	0.543	(-6.139 ; 7.226)	1.000
Parks counts in 1500m² network walkable street buffers	-0.345	(-2.616;1.926)	1.000	0.106	(-2.08 ; 2.292)	1.000
Beaches counts in 1500m² network walkable street buffers	6.892	(-5.036;18.821)	1.000	5.266	(-6.016 ; 16.548)	1.000

Abbreviations: POS, public open spaces; LTPA, leisure time physical activity; ß, non-standardized coefficient; CI, confidence interval. ß indicates change in total LTPA Metabolic equivalent task per minutes per day (METs min/d) per increment (in 100m2 or count) in access to public open spaces (POS). Model 1: unadjusted linear regression. Model 2: linear regression adjusted by individual-level covariates (sex, age, body mass index, educational level and smoking habits). Due the used of multiple comparisons we applied the Bonferroni post-hoc correction on both models.

Table S3. Association between access to POS and light LTPA.

Predictor variable	Model 1			Model 2		
	ß	CI	¶ P	ß	CI	¶ P
Distance to the nearest sports facility (100m)	0.756	(-1.536 ; 3.048)	1.000	0.181	(-2.098 ; 2.46)	1.000
Distance to the nearest Park (100m)	-3.44	(-8.288 ; 1.408)	1.000	-4.378	(-9.147 ; 0.392)	0.728
Distance to the nearest beach (100m)	-0.61	(-1.202 ; -0.018)	0.439	-0.642	(-1.23 ; -0.054)	0.329
Distance to the coast (100m)	-0.592	(-1.252 ; 0.068)	0.796	-0.703	(-1.364 ; -0.042)	0.377
Sports facility areas in 500m² network walkable street buffers (100m²)	-0.302	(-0.599 ; -0.006)	0.828	-0.322	(-0.615 ; -0.028)	0.576
Parks areas in 500m² network walkable street buffers (100m²)	0.049	(-0.059 ; 0.157)	1.000	0.073	(-0.034 ; 0.179)	1.000
Beaches areas in 500m² network walkable street buffers (100m²)	0.022	(-0.231 ; 0.275)	1.000	-0.005	(-0.253 ; 0.244)	1.000
Sports facility areas in 1000m² network walkable street buffers (100m²)	-0.074	(-0.189 ; 0.04)	1.000	-0.079	(-0.192 ; 0.034)	1.000
Parks areas in 1000m² network walkable street buffers (100m²)	0.025	(-0.006 ; 0.055)	1.000	0.029	(-0.002 ; 0.059)	1.000
Beaches areas in 1000m² network walkable street buffers (100m²)	0.016	(-0.09 ; 0.122)	1.000	0.003	(-0.101 ; 0.107)	1.000
Sports facility areas in 1500m² network walkable street buffers (100m²)	-0.07	(-0.132 ; -0.008)	0.486	-0.067	(-0.129 ; -0.006)	0.576
Parks areas in 1500m² network walkable street buffers (100m²)	0.018	(0.004 ; 0.031)	0.180	0.02	(0.006 ; 0.034)	0.072
Beaches areas in 1500m² network walkable street buffers (100m²)	-0.001	(-0.074 ; 0.072)	1.000	-0.01	(-0.082 ; 0.061)	1.000
Sports facility counts in 500m² network walkable street buffers	-12.065	(-26.015 ; 1.884)	1.000	-9.345	(-23.196 ; 4.507)	1.000
Parks counts in 500m² network walkable street buffers	4.208	(-1.531 ; 9.948)	1.000	5.564	(-0.085 ; 11.214)	0.972
Beaches counts in 500m² network walkable street buffers	3.058	(-11.334 ; 17.45)	1.000	-0.043	(-14.232 ; 14.145)	1.000
Sports facility counts in 1000m² network walkable street buffers	-1.802	(-8.763 ; 5.159)	1.000	-0.875	(-7.736 ; 5.985)	1.000
Parks counts in 1000m² network walkable street buffers	1.765	(-0.337 ; 3.867)	1.000	2.102	(0.018 ; 4.185)	0.882
Beaches counts in 1000m² network walkable street buffers	2.608	(-5.252 ; 10.468)	1.000	1.346	(-6.392 ; 9.084)	1.000
Sports facility counts in 1500m² network walkable street buffers	-0.514	(-4.001 ; 2.974)	1.000	-0.056	(-3.504 ; 3.391)	1.000
Parks counts in 1500m² network walkable street buffers	0.766	(-0.358 ; 1.891)	1.000	0.986	(-0.138 ; 2.11)	1.000
Beaches counts in 1500m² network walkable street buffers	2.971	(-2.95 ; 8.893)	1.000	2.224	(-3.598 ; 8.046)	1.000

Abbreviations: POS, public open spaces; LTPA, leisure time physical activity; ß, non-standardized coefficient; CI, confidence interval. ß indicates change in light LTPA Metabolic equivalent task per minutes per day (METs min/d) per increment (in 100m, 100m2 or count) in access to public open spaces (POS). Model 1: unadjusted linear regression. Model 2: linear regression adjusted by individual-level covariates (sex, age, body mass index, educational level and smoking habits). Due the used of multiple comparisons we applied the Bonferroni post-hoc correction on both models.

Table S4. Association between access to POS and moderate-vigorous LTPA.

Predictor variable	Model 1			Model 2		
	ß	CI	¶ P	ß	CI	¶ P
Distance to the nearest sports facility (100m)	4.971	(0.944 ; 8.999)	0.160	4.286	(0.343 ; 8.228)	0.337
Distance to the nearest Park (100m)	20.102	(11.726 ; 28.478)	0.000**	17.773	(9.621 ; 25.924)	0.000**
Distance to the nearest beach (100m)	0.053	(-0.999 ; 1.104)	1.000	0.021	(-1.007 ; 1.049)	1.000
Distance to the coast (100m)	0.377	(-0.794 ; 1.547)	1.000	0.232	(-0.923 ; 1.387)	1.000
Sports facility areas in $500m^2$ network walkable street buffers ($100m^2$)	0.053	(-0.473 ; 0.579)	1.0000	0.006	(-0.508 ; 0.52)	1.000
Parks areas in $500m^2$ network walkable street buffers ($100m^2$)	-0.157	(-0.347 ; 0.033)	1.0000	-0.12	(-0.306 ; 0.065)	1.000
Beaches areas in $500m^2$ network walkable street buffers ($100m^2$)	-0.082	(-0.53 ; 0.366)	1.0000	-0.123	(-0.556 ; 0.31)	1.000
Sports facility areas in $1000m^2$ network walkable street buffers ($100m^2$)	0.112	(-0.091 ; 0.314)	1.0000	0.082	(-0.115 ; 0.28)	1.000
Parks areas in $1000m^2$ network walkable street buffers ($100m^2$)	-0.011	(-0.065 ; 0.043)	1.0000	-0.006	(-0.06 ; 0.047)	1.000
Beaches areas in $1000m^2$ network walkable street buffers ($100m^2$)	0.049	(-0.138 ; 0.236)	1.0000	0.028	(-0.153 ; 0.21)	1.000
Sports facility areas in $1500m^2$ network walkable street buffers ($100m^2$)	0.023	(-0.087 ; 0.133)	1.0000	0.029	(-0.078 ; 0.137)	1.000
Parks areas in $1500m^2$ network walkable street buffers ($100m^2$)	-0.014	(-0.039 ; 0.01)	1.0000	-0.011	(-0.035 ; 0.013)	1.000
Beaches areas in $1500m^2$ network walkable street buffers ($100m^2$)	0.03	(-0.099 ; 0.159)	1.0000	0.018	(-0.107 ; 0.143)	1.000
Sports facility counts in $500m^2$ network walkable street buffers	-9.259	(-33.989 ; 15.47)	1.0000	-7.417	(-31.548 ; 16.714)	1.000
Parks counts in $500m^2$ network walkable street buffers	-10.023	(-20.151 ; 0.104)	0.9558	-8.124	(-17.962 ; 1.715)	1.000
Beaches counts in $500m^2$ network walkable street buffers	10.963	(-14.466 ; 36.391)	1.0000	6.013	(-18.657 ; 30.684)	1.000
Sports facility counts in $1000m^2$ network walkable street buffers	-1.709	(-14.018 ; 10.6)	1.0000	-0.837	(-12.77 ; 11.096)	1.000
Parks counts in $1000m^2$ network walkable street buffers	-1.788	(-5.512 ; 1.936)	1.0000	-1.786	(-5.423 ; 1.851)	1.000
Beaches counts in $1000m^2$ network walkable street buffers	6.292	(-7.599 ; 20.182)	1.0000	4.552	(-8.901 ; 18.005)	1.000
Sports facility counts in $1500m^2$ network walkable street buffers	-0.034	(-6.2 ; 6.132)	1.0000	0.589	(-5.407 ; 6.585)	1.000
Parks counts in $1500m^2$ network walkable street buffers	-1.115	(-3.104 ; 0.875)	1.0000	-0.883	(-2.843 ; 1.076)	1.000
Beaches counts in $1500m^2$ network walkable street buffers	3.924	(-6.55 ; 14.397)	1.0000	3.045	(-7.084 ; 13.173)	1.000

Abbreviations: POS, public open spaces; LTPA, leisure time physical activity; ß, non-standardized coefficient; CI, confidence interval. ß indicates change in moderate-vigorous LTPA Metabolic equivalent task per minutes per day (METs min/d) per increment (in 100m, 100m2 or count) in access to public open spaces (POS). Model 1: unadjusted linear regression. Model 2: linear regression adjusted by individual-level covariates (sex, age, body mass index, educational level and smoking habits). Due

the used of multiple comparisons we applied the Bonferroni post-hoc correction on both models. ¶ P Values with asterisks are statistically different from baseline by Bonferroni post-hoc test ($P < 0.05$): *$P < 0.05$; **$P < 0.01$.
